# Musical and Bodily Predictors of Mental Effort in String Quartet Music: An Ecological Pupillometry Study of Performers and Listeners

**DOI:** 10.3389/fpsyg.2021.653021

**Published:** 2021-06-28

**Authors:** Laura Bishop, Alexander Refsum Jensenius, Bruno Laeng

**Affiliations:** ^1^RITMO Centre for Interdisciplinary Studies in Rhythm, Time and Motion, University of Oslo, Oslo, Norway; ^2^Department of Musicology, University of Oslo, Oslo, Norway; ^3^Department of Psychology, University of Oslo, Oslo, Norway

**Keywords:** pupillometry, mental effort, music performance, music listening, musical expression, arousal

## Abstract

Music performance can be cognitively and physically demanding. These demands vary across the course of a performance as the content of the music changes. More demanding passages require performers to focus their attention more intensity, or expend greater “mental effort.” To date, it remains unclear what effect different cognitive-motor demands have on performers' mental effort. It is likewise unclear how fluctuations in mental effort compare between performers and perceivers of the same music. We used pupillometry to examine the effects of different cognitive-motor demands on the mental effort used by performers and perceivers of classical string quartet music. We collected pupillometry, motion capture, and audio-video recordings of a string quartet as they performed a rehearsal and concert (for live audience) in our lab. We then collected pupillometry data from a remote sample of musically-trained listeners, who heard the audio recordings (without video) that we captured during the concert. We used a modelling approach to assess the effects of performers' bodily effort (head and arm motion; sound level; performers' ratings of technical difficulty), musical complexity (performers' ratings of harmonic complexity; a score-based measure of harmonic tension), and expressive difficulty (performers' ratings of expressive difficulty) on performers' and listeners' pupil diameters. Our results show stimulating effects of bodily effort and expressive difficulty on performers' pupil diameters, and stimulating effects of expressive difficulty on listeners' pupil diameters. We also observed negative effects of musical complexity on both performers and listeners, and negative effects of performers' bodily effort on listeners, which we suggest may reflect the complex relationships that these features share with other aspects of musical structure. Looking across the concert, we found that both of the quartet violinists (who exchanged places halfway through the concert) showed more dilated pupils during their turns as 1st violinist than when playing as 2nd violinist, suggesting that they experienced greater arousal when “leading” the quartet in the 1st violin role. This study shows how eye tracking and motion capture technologies can be used in combination in an ecological setting to investigate cognitive processing in music performance.

## 1. Introduction

Music performance is a cognitively demanding activity that requires many processes to be carried out in parallel, including overt motor production, covert processing of musical information, monitoring of musical output, and monitoring of audience responses (Bishop and Keller, 2021)^1^. There are additional demands during ensemble performance; for example, performers must divide attention between their own playing and their co-performers' playing (Keller, 2001).

A hallmark of skilled performance is the ability to manage cognitive resources effectively so that accuracy, expressivity, and (in ensemble settings) coordination are maintained. Performances by skilled musicians may seem rather effortless to audience members, but they actually draw on a combination of effortful and automatic processes. These processes involve performers' anticipation of each other's playing (including more effortful imagery and simulation and more automatic melodic expectancies), adaptation to each other's playing (including more effortful period correction and more automatic phase correction), and control of attention (including more effortful directed listening and more automatic passive monitoring).

Skilled performers are able to prioritize one process over another, focusing attention on the prioritized process while non-prioritized processes run automatically. To facilitate attention regulation, musicians may identify landmarks in the music that can serve as cues to attention, drawing their focus to specific technical or expressive processes (Chaffin and Logan, 2006; Chaffin et al., 2010). Less skilled performers may lack attention regulation abilities and, as a result, distribute attention non-optimally—for instance, by focusing on their own playing when they should be listening to their co-performers, or sacrificing expressivity to focus on note accuracy.

In this study, we examine the relationships between the cognitive-motor demands of string quartet performance and attention fluctuations in both performers (Experiment 1) and listeners (Experiment 2). We used the psychophysiological method of pupillometry to gauge changes in the intensity of attention (Kahneman, 1973; Laeng and Alnæs, 2019). In Experiment 1, we collected pupillometry, motion capture, and audio-video data from a string quartet as they performed selections of classical repertoire in our lab in rehearsal and concert/exam conditions. In Experiment 2, we collected pupil data from musically-trained listeners as they heard recordings of the quartet's concert performance. We then analysed how changes in performers' and listeners' pupil diameters related to features of the performers' physical performance (e.g., quantity of motion) and features of the music (e.g., tonal tension and expressivity). In the sections below, we develop some predictions for how these features draw on attention.

### 1.1. Pupil Size as an Index of Mental Effort

Pupil size is commonly used as an index of attention and mental effort in cognitive tasks (van der Wel and van Steenbergen, 2018; Laeng and Alnæs, 2019). Pupil size is tightly coupled to the release of norepinephrine by the locus coeruleus, which modulates attention and cognitive arousal (e.g., Sara, 2009; Alnæs et al., 2014; Joshi et al., 2016). Pupil dilations occur reliably as part of an orienting response to salient, attention-grabbing stimuli across modalities (Murphy et al., 2016; Marois et al., 2018) or whenever an individual is focused on a challenging task (Laeng et al., 2011). These dilations, described as psychosensory pupil responses (Mathôt, 2018), can be sampled at a fine resolution with modern eye-trackers and act as a gauge of moment-to-moment attention fluctuations.

A number of factors may contribute to how intensely attention is focused at any given moment during a music performance, including the complexity of the music and how technically difficult it is to play. This intensity of cognitive processing is referred to as “mental effort” regardless of the type of task being performed (Kahneman, 1973). Studies of mental effort have shown that pupil dilations occur during complex tasks such as comprehending sentences with higher linguistic complexity (Just et al., 2003), during tasks carried out under interference (O'Shea and Moran, 2019), and when working memory load is high (Kahneman and Beatty, 1966; Klingner et al., 2011; see also Zekveld et al., 2018). Conversely, pupil constriction occurs during periods of distraction and mind wandering (Konishi et al., 2017). Individual differences in cognitive abilities such as working memory capacity also contribute to attention control (Unsworth and Robison, 2017; Endestad et al., 2020).

In a musical context, patterns of pupil dilations reflect listeners' entrainment with musical rhythms (Fink et al., 2018) and listeners' attention to deviations from strict rhythmic regularity. These deviations are referred to as “microtiming” in the context of groove-based jazz music (Skaansar et al., 2019), but are also a common feature of expressively-performed music in many traditions. A pupil response is also observed when listeners hear pitches that deviate from an established tonal context, or in general, when they are surprising (Liao et al., 2016; Bianco et al., 2020). Musical features that capture attention tend to do so reliably across listeners who are familiar with the musical tradition, and as a result, similar patterns of pupil dilation occur among listeners who hear the same musical material (Kang and Wheatley, 2015; Kang and Banaji, 2020).

### 1.2. Mental Effort and Musical Complexity

Music performance and listening involves continual processing of tonal and timing information (Huron, 2006). Music that violates listeners' expectations for tonality or timing can trigger an increase in mental effort. Fluctuations in mental effort might also occur in response to the complexity of the music that is performed. Musical complexity can be described as a property of a musical stimulus that increases as the degree of uncertainty or unpredictability of pitch, timing, and other features increases. For example, a piece in which many pitch classes have an equal probability of occurring could be deemed more complex than a piece in which few pitch classes are more probable. The complexity of a musical stimulus can also be said to relate to the amount of change that occurs over time (Mauch and Levy, 2011) or the number of events per part or layer.

From a psychological perspective, it is important to account for how listeners perceive music when assessing its complexity. Listeners' perceptions of complexity are influenced by their long-term musical knowledge, which develops through their exposure to different kinds of music. Studies have shown an inverted U-shaped relationship between complexity and preference (Burke and Gridley, 1990; Gordon and Gridley, 2013), and a relationship between working memory capacity and preference for complexity, mediated by musical training (Vuvan et al., 2020). Marin and Leder (2013) found that arousal mediated a relationship between musical complexity and listeners' ratings of pleasantness.

For the present study, we estimated harmonic complexity, a subcomponent of musical complexity, using a measure of harmonic tension. Harmonic tension and complexity are overlapping phenomena. Both are aggregate constructs that draw on a combination of psychoacoustic features including tonal, temporal, and timbral information. We chose to focus on the tonal component because tonality is particularly relevant in the repertoire that was performed by our quartet. In Western tonal music, moment-to-moment changes in harmonic complexity contribute to listeners' perceptions of harmonic tension, which is usually defined in qualitative terms, with increasing tension described as a feeling of rising intensity and decreasing tension described as a feeling of resolution.

One of our measures of harmonic complexity, “Cloud diameter,” derives from the spiral array model of tonality that was proposed for tonal music by Chew (2000). This model is a 3D extension of the circle of fifths, in which pitch classes that are tonally close (e.g., a perfect fifth) are in close spatial proximity to each other. Cloud diameter is computed from musical scores. It represents the tonal distance within a cluster of notes and is given in terms of the spatial distance between their pitch classes in the spiral array (Herremans and Chew, 2016). We predicted that increased Cloud diameter (i.e., increased dissonance) would require increased mental effort to process, resulting in greater pupil size among performers and listeners.

We also obtained ratings of changes in harmonic complexity throughout the pieces from the quartet, which they gave individually per bar for their own parts. These ratings were expected to correlate moderately with Cloud diameter, and were predicted to relate to increased pupil diameter in both performers and listeners.

### 1.3. Bodily Effort and Mental Effort

Playing music is physically effortful. The processes of carrying out and controlling body motion involves some mental effort. It is therefore important to consider bodily effort when assessing mental effort in performers. In sports, pupil dilations have been shown to occur at the onset of “quiet eye”—the prolonged fixation that expert athletes make on a target immediately prior to initiating a goal-directed action—suggesting heightened mental effort is involved in action preparation (Vickers, 2009; Campbell et al., 2019; Piras et al., 2020). However, the relationship between sustained bodily effort and mental effort generally remains unclear.

For musicians, the physical demands of performance can be described in terms of two components: physical force (or exertion) and control. Force is an important dimension of bodily effort for acoustic instrument performers, as it is the primary means of controlling sound intensity (Olsen and Dean, 2016). Control is central to effective playing technique and relates to how precisely a performer can achieve their intended timing, intonation, timbre, and dynamic level (Palmer, 1997; Bishop and Goebl, 2017). These components of bodily effort can vary independently; for example, performing rapid notes at a low dynamic level requires little force but high control, and can be technically demanding.

In a study by Zénon et al. (2014), pupil size related to the intensity of bodily effort (the amount of force exerted in a power grip task) as well as to participants' perceptions of effort. van der Wel and van Steenbergen (2018) additionally argue that task demands and the amount of effort that participants actually invest in a task can diverge. A recent study of mental effort in imagined and overt piano playing showed a divergence between task demands and measures of mental effort when the difficulty of the task exceeded the capacities of the participants (e.g., when complex movements had to be imagined at a fast tempo; or when imagery had to be carried out with interference; O'Shea and Moran, 2019). Interestingly, even though pupils constricted during the most difficult conditions in this study, participants reported increased mental effort, indicating a dissociation between pupil size and perceptions of effort.

In contrast, a recent study by Endestad et al. (2020), also using pupillometry, showed a clear relationship between pupil size and degrees of mental effort during overt and imagined piano playing for a professional pianist as well as for listeners. This study also showed, using fMRI, differences in locus ceruleous activity for the same pianist as she played (in the scanner) two pieces of different difficulty.

The bodily effort that is involved in playing music affects listeners' experiences of the music as well as performers'. The embodied music cognition framework posits that music perception is a body-based cognitive process that draws on listeners' motor systems in various ways (Maes et al., 2014). Some supporting evidence comes from neuroimaging studies, which have shown that listening to music activates motor circuits, even when the listener makes no overt motion (e.g., Abrams et al., 2013; Gordon et al., 2018). The patterns of activity across motor regions may be especially similar between performers and listeners when they share instrument-specific expertise (Haueisen and Knösche, 2001; Taylor and Witt, 2014).

The activation of motor circuits during music listening may allow listeners to covertly simulate features of the actions that were involved in playing the music (Wilson and Knoblich, 2005; Repp and Knoblich, 2007). This real-time simulation of music performance actions may help listeners to generate predictions that shape their perception of the music. Motor activation may also occur at a more general level, allowing for simulation of features of actions that listeners have no experience in performing, or remapping of action features to familiar action sequences (e.g., allowing a listener to covertly sing along with a melody played by a violin, despite having no violin-playing experience; Godøy et al., 2005; Eitan and Timmers, 2010; Maes et al., 2014; Kelkar and Jensenius, 2018). Motor activation during music listening may furthermore help listeners to construct expressive interpretations of the music they hear and relate to the performer(s) on an emotional level (Molnar-Szakacs et al., 2011; Olsen and Dean, 2016).

In the current study, we assessed the effect of bodily effort on pupil changes during string quartet performance and listening. We predicted that the bodily effort that performers invested in their playing would engage increased mental effort. We also predicted that the bodily effort that listeners heard in recordings of the quartet's performances would engage increased mental effort, especially in the case of string musicians, who would be most familiar with the sound-producing actions involved in quartet playing. Bodily effort was operationalized in terms of several different measures, which we selected to capture different aspects of the physical demands of playing a stringed instrument. These included measures of overt head and arm motion, acoustic intensity (taken as a correlate of physical force), and the performers' subjective, per-bar ratings of technical difficulty.

### 1.4. Expressivity, Arousal, and Mental Effort

Attention-related modulations of pupil size also reflect changes in arousal. For both performing musicians and listeners, local fluctuations in arousal can occur in relation to expressive changes in the music (Schubert, 2004; Lundqvist et al., 2009; Egermann et al., 2015). Musical expression is a construct that arises from interactions between different musical parameters, including pitch, timing, dynamics, timbre, and various body features, among others (Juslin, 2003; Jensenius et al., 2010; Cancino-Chacón et al., 2017). For music in the Western classical tradition, expressivity is to a large extent tied to certain key structural features that are given in a score (Palmer, 1997). Performers may interpret these features in different ways, thus producing performances that are expressively distinct.

One component of musical expression is emotional expression (Juslin, 2003). Music can be emotionally expressive in different ways, including through extramusical associations and through perceptual expectations that arise from familiar harmonic relationships (Egermann et al., 2013; Pearce, 2018). The emotional qualities of music are commonly described in terms of arousal and valence (e.g., Schubert, 2004). Many of the basic emotions that people report associating with music can be readily placed in a two-dimensional space that crosses arousal with valence (e.g., happiness, peacefulness, fear, etc.), though this is not the case for some more complex emotions, such as being moved (*kama muta*) or awe, which contain elements of seemingly contradictory emotional states (e.g., awe is described as including aspects of both sadness and joy; Konecni, 2005; Menninghaus et al., 2015; Zickfeld et al., 2019).

A few studies have investigated the relationship between pupil size and emotional arousal in either music performers or listeners. Gingras et al. (2015) showed a positive relationship between pupil dilation and ratings of emotional arousal and tension in listeners who heard brief (6-s) excerpts of Romantic-style piano trios. In another study, pupil dilations were shown to occur in close temporal proximity to listeners' reported chills (i.e., peak emotional experiences; Laeng et al., 2016). In a study investigating the arousal elicited by vocal vs. instrumental melodies, listeners displayed a more dilated pupil when hearing vocal melodies than when hearing the same melodies played on a piano, suggesting that the human voice is treated as a “privileged signal” (Weiss et al., 2016). In the same study, listeners also showed a more dilated pupil when hearing familiar melodies than when hearing novel melodies.

The current study contributes to this literature with an investigation of how local fluctuations in expressivity relate to pupil size. Expressivity was quantified through performers' per-bar ratings of expressive/interpretive difficulty. It should be noted that we did not ask for performers' ratings of music-related arousal or expressive intensity, although we expect that these factored into the ratings that they gave (see Methods for our exact wording). Their ratings may also reflect their judgements of musical complexity and technical difficulty, which also contribute to how readily performers realize their expressive goals. In short, our measure of expressive difficulty probably constitutes a higher-order indication of the performers' relationships with the music. We predicted that higher ratings of expressive difficulty would correspond to higher emotional arousal and, correspondingly, larger pupil size for both performers and listeners.

### 1.5. Arousal and Attention Regulation During Music Performance

Changes in performers' arousal can occur across relatively long timeframes (e.g., across the course of a concert). These changes occur in addition to the local fluctuations that relate to musical expression, and can be assessed with pre-trial “baseline” pupil measurements. For performers, baseline levels of arousal are likely to depend on the conditions surrounding their performance (e.g., who is in the audience, how well-prepared the performers are, etc.) and their individual response to those conditions. Elevated arousal prior to public performance is common, and often associated with performance anxiety (Kenny, 2011). Performances are given optimally under moderate levels of arousal (Papageorgi et al., 2007). Physiological correlates of autonomic arousal, including increased heart rate, increased motor excitability, and sweating, can themselves be detrimental to performance, impairing fine motor control and increasing the bodily effort that is required to maintain technical accuracy. Musicians may have to deviate from their practiced playing technique in order to compensate and maintain control of their movements (Yoshie et al., 2009). This makes performance more difficult and adds to musicians' mental workload. Absorption (sometimes described in terms of flow) is noted to emerge predominately under moderate levels of arousal (Peifer et al., 2014; Vroegh, 2019).

Effective attention regulation is also thought to require a moderate level of arousal (Unsworth and Robison, 2017). Lenartowicz et al. (2013) suggest that high and low levels of arousal pave the way for different types of distractibility. They posit a “landscape” of attention control states based on crossing high and low arousal levels with internal and external attention orientations. If arousal is low, and internal focus can lead to mind wandering and zoning out, while an external focus leads to behaviour comprising predominately automatic responses to salient stimuli. If arousal is high, an inwards focus can result in mind-racing, while an external focus leads to excessive and nondiscriminating responses to both relevant and irrelevant stimuli.

For ensembles like a classical string quartet, baseline arousal levels might be expected to differ between performers according to their individual roles in the ensemble. Traditionally, the 1st violinist is the leader of the group. This is particularly the case for repertoire from the classical period (e.g., including works by Haydn and Mozart), where the 2nd violinist, violist, and cellist typically have more supporting roles. The 1st violinist is also often responsible for giving cues to the other musicians to help keep the group together. While string quartets may operate more democratically in other ways (e.g., jointly making interpretative decision), anecdotal evidence suggests that some 1st violinists feel heightened stress in performance due to their leadership role (Davidson and Good, 2002). If this is heightened stress occurs, it is likely reflected in the 1st violinist's pupil dilations. We predicted that the 1st violinist would show more dilated pupils than the other musicians. We also predicted that the musicians would show more dilated pupils in the baseline measurement taken just before the start of the concert performance than in the baseline measurements taken before the rehearsal performances, earlier in the same recording session.

### 1.6. Current Study

This study made a novel assessment of how musical complexity, bodily effort, expressive difficulty, and situational factors (including rehearsal vs. concert setting, piece order, and musical role) contribute to mental effort in performers and listeners of string quartet music. In Experiment 1, we invited a student string quartet from a local music academy to record some performances of their current repertoire at our lab. The quartet gave five performances of an excerpt from one of their pieces in rehearsal conditions (i.e., with no audience). We manipulated the configuration of the quartet across rehearsal performances in order to partially or completely disrupt visual communication between musicians. Bishop et al. (2021)^2^ reports on how these manipulations affected interperformer communication during the rehearsal performances. The current paper does not consider these manipulations further.

Following the rehearsal performances, the quartet played the full set of pieces for a live audience (which included an examiner) in a concert/exam condition. We collected pupillometry, gaze, motion capture, and audio/video data from the musicians. The performers later individually provided per-bar ratings of Harmonic complexity, Technical difficulty, and Expressive difficulty. In Experiment 2, we collected pupillometry data from 16 trained musicians as they listened to recordings of the quartet's concert performance.

**Figure 1:**
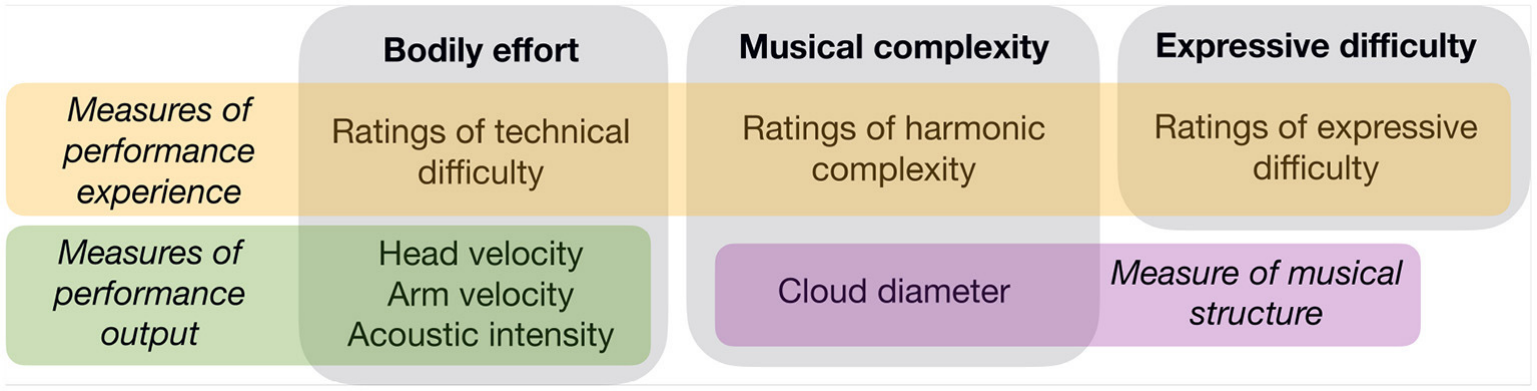
List of predictors relating to bodily effort, musical complexity, and expressive difficulty.

Musical complexity, bodily effort, and expressive difficulty were subdivided into a combination of predictors that included ratings provided by the performers and measurements of score information and performance data (Figure 1). With this combination of predictors, we aimed to capture the effects of perceived effort, allocated effort, and task difficulty. The overarching prediction was that subjective and objective measures of both musical complexity and bodily effort would relate to increased pupil dilations. We predicted a similar pattern of results for performers and listeners, although we expected a larger effect of predictors relating to performers' bodily effort on performers than on listeners.

As predictors relating to musical complexity, we included Cloud diameter as a measure of harmonic tension, and performers' ratings of Harmonic complexity. As predictors relating to bodily effort, we included quantity of head and arm motion, energy (acoustic intensity) of the musical sound signal, and performers' ratings of technical difficulty. For string musicians, head motion is not directly involved in sound production, but may be representative of musicians' expressive engagement with the music (see Glowinski et al., 2013a,b) and communication with co-performers (Bishop and Goebl, 2018)^2^. Sound intensity can be considered a correlate of physical force, as more forceful movement is required to produce higher-intensity audio signals on string instruments. Our measure of expressive difficulty, which we posited incorporated aspects of arousal, musical complexity, and technical difficulty, constituted ratings provided by the performers.

We additionally made several predictions relating to situational factors, which were tested in Experiment 1. First, we predicted that arousal would be greater at the start of the concert than at the start of the rehearsal period. We also predicted that during the course of the concert, some global changes in performers' tonic arousal would occur as their initial anxiety reduces. We tested for differences in mean pupil size between the four pieces that the quartet played in the concert, expecting that a gradual decline in arousal would occur. We also predicted that levels of arousal would be tied to the different musical roles of the quartet members. The violinists switched roles halfway through the concert, so that each played the 1st violin part for two of the four pieces. This gave us an opportunity to test the prediction that the 1st violinist would have heightened arousal due to their leadership role.

## 2. Experiment 1: Mental Effort in String Quartet Rehearsal and Concert Performance

### 2.1. Participants

A student string quartet from a local music academy took part in the experiment (1 female, 3 males; ages 19–20; 13–16 years of music training). They had established themselves as a group 6 months prior to the experiment, but had occasionally played together in various ensembles before this, having attended the same music school as children. The 2nd violinist and cellist had played together in a quartet for 11 years. At the time of the experiment, the quartet had been rehearsing the Haydn piece for 3 months and the Debussy piece for 2 months. The musicians all provided written informed consent.

### 2.2. Materials and Equipment

**Figure 2:**
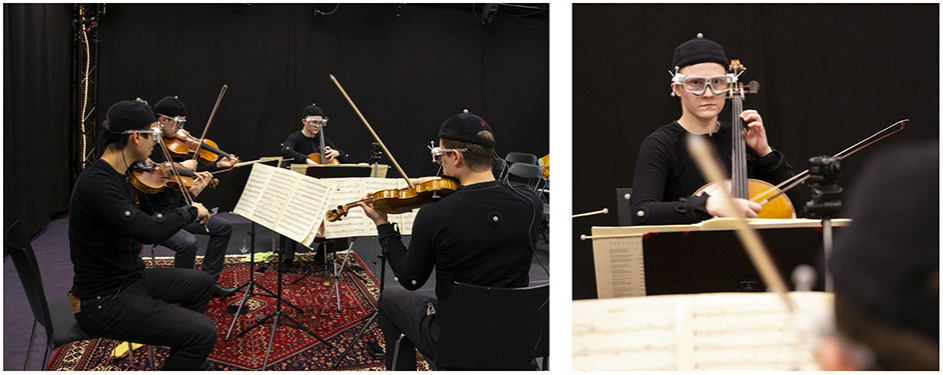
Photos showing the eye-tracking glasses and locations of body markers on the musicians. Photo credit: Annica Thomsson.

Motion data were recorded using a Qualisys system with 12 Oqus 300 cameras (performances 1–6) and an OptiTrack system with 8 Flex 13 cameras (performance 7); see Figure 2^3^. The musicians wore jackets and caps with six reflective markers attached (1 on the head, 1 on the upper back, and 2 on each arm above and below the elbow). Marker positions were sampled at 120 Hz. Pupil and gaze data were collected using SMI Wireless Eye Tracking Glasses, which recorded at 60 Hz. To synchronize motion and eye data, we recorded an audiovisual signal using a clapperboard with 2 reflective markers affixed at the start of each performance. Recordings were aligned retrospectively from this point. Additional details on our equipment set-up relating to measures that are not reported here are given by^2^.

The experiment was carried out in our lab, which has moderately bright lighting and black curtains covering the walls and windows. To avoid adding noise to the pupillometry data, we did not use any spotlights or stage lighting.

During the rehearsal performances, the musicians played the first 68 bars of the first movement of the String Quartet in B-flat major, Op. 76, No. 4, by Haydn. For the concert, they played the full first and second movements of this work as well as the full first and second movements of the String Quartet in G minor, Op. 10, by Debussy. Hereafter, we will refer to these pieces as Haydn I and II and Debussy I and II.

### 2.3. Procedure

The musicians warmed up briefly and were then positioned for the first performance. They completed a 1-min baseline pupil recording before playing, for which they were instructed to sit still and focus their gaze on a single score note. The performance was then recorded, and the musicians were repositioned for the next condition. Baseline recordings were made every time the musicians were repositioned. Following the Replication-rehearsal, we paused so that the musicians could take a break and the lab could be set up with audience seating for the concert. A final baseline and then the concert were recorded. In total, including setup and breaks, the experiment took around 4 h.

In the weeks following the recording session, the musicians made per-bar ratings of perceived difficulty for the music that they played during the concert. They rated the music on three measures, using a scale of 1—7: technical difficulty (how technically challenging was the bar to play?); expressive/interpretative difficulty (how difficult was it to express the intended meaning, idea, or emotion?); and harmonic tension/complexity (how harmonically complex or tense was the material in the bar?). The rating task was done separately and individually and the musicians submitted scanned and rated copies of their scores when they had finished.

### 2.4. Analysis

#### 2.4.1. Preprocessing of Pupil, Motion, and Musical Data

##### 2.4.1.1. Pupil Data

We used binocular pupil diameters (in mm) for our analysis, which we obtained by averaging the values that were recorded for left and right eyes. We used binocular rather than monocular values in order to minimize any effects of any outliers that might occur in one eye or the other (especially in moments where participants were looking at more extreme angles). A multi-step procedure was developed for cleaning and filtering binocular pupil data. First, to eliminate blinks, we discarded any observations where the recorded diameter was more than 2 standard deviations below the mean diameter for the trial. We found that it was also necessary to discard some non-zero values at the edges of the “gaps” that occurred because of blinks, where the eye was captured partially closed. These values were identified on the basis of velocity, which was calculated as the first derivative (rate of change) of the series of pupil diameters. Observations where the velocity of diameter change was more than 2 standard deviations from the mean velocity of the trial were discarded. A Savitzky-Golay filter was used to smooth the remaining data (order = 3, window = 11), and gaps in the data were filled using a linear interpolation. Finally, blinks were removed from baseline data, and smoothed performance data were calculated as differences from mean baseline diameters.

Since the ratings provided by the performers were given per bar, we downsampled the processed pupil data to obtain an average diameter per bar. To do this, we interpolated a series of bar onset times using the audio recordings. Bars were assumed to be evenly spaced in time. Although we acknowledge that this introduces some imprecision into our alignments, a more precise audio-to-score mapping would have been a substantial task and beyond the scope of this project. Pupil data and bar numbers were then aligned based on their timestamps, and we calculated an average pupil diameter per bar. These averaged, per-bar diameters were analysed in the linear mixed effects models (see below).

##### 2.4.1.2. Motion Data

Head and arm data were used for the analyses presented here. We chose to focus on *velocity* rather than a higher order kinematic feature (e.g., smoothness) because velocity provides a more direct measure of the mechanical energy that is expended by a performer and is related to momentum. Smoothed velocities were derived using a Savitzky-Golay filter (order = 3, window = 41; “savitzkyGolay” function from the “prospectr” package in R, which optionally outputs smoothed derivatives of the input data). The norm of smoothed 3D velocities was then computed. Using the bar onset times that we describe above (see Pupil data), we aligned the motion data with bar numbers based on their timestamps. “Quantity of motion” (QoM) was then calculated as the sum of velocities per bar of each piece.

##### 2.4.1.3. Audio Data

Root mean square (RMS) values were extracted from audio recordings as a measure of acoustic intensity. This was done in Python using the package Madmom (Böck et al., 2016), with a frame size of 2048 samples and 50% overlap. RMS curves were smoothed using a convolution-based method, with a Hamming window of 50 samples. The resulting RMS values were averaged per bar for the linear mixed effects model analysis. Hereafter we refer to these values as “sound level.”

##### 2.4.1.4. Cloud Diameter

Cloud diameters were calculated for the score of each piece in Python, using the package Partitura (Grachten et al., 2019), at increments of 1 bar. The algorithm requires scores in musicXML format. We obtained MIDI files for all of the pieces online^4^, hand-corrected them for pitch spelling (with reference to the scores that were used by the quartet), and converted them to musicXML in MuseScore^5^. Output Cloud diameters are given in units of a perfect fifth in Chew (2000)'s spiral array.

#### 2.4.2. Linear Mixed Effects Modelling of Pupil Diameter

Linear mixed effects models (LMMs) were used to test the contribution of predictors relating to musical complexity, bodily effort, and expressive difficulty to baseline-normalized pupil diameter. This was done using the “glmmTRB” package in R.

We tested two models, one which included head motion as an index of bodily effort, and one that included arm motion instead. Model 2 (with arm motion) included fewer data points than Model 1 (with head motion) because some of the arm markers, especially for the 2nd violinist and violist, were not as well tracked as the head markers were. As a result, we could only include arm data for 1–2 pieces for these performers. Nonetheless, it was important to consider arm motion as a measure of bodily effort because it is directly tied to sound production.

- **Model 1** included seven fixed effects: quantity of Head motion, Sound level, Technical difficulty ratings, Cloud diameter, Harmonic complexity ratings, and Expressive difficulty ratings.
- **Model 2** included quantity of Arm motion (*instead of* Head motion), Sound level, Technical difficulty ratings, Cloud diameter, Harmonic complexity ratings, and Expressive difficulty ratings.

For both models, musician and performance were included as crossed random effects. Since our predicted variable constituted time series data, we also specified an autocorrelation structure (order = 1) with time (in bars) as a covariate and the same grouping structure as our random effects.

The formulation of Model 1 was as follows:
*Pupil size* ~ *Cloud diameter* + *Harmonic complexity ratings* + *Head motion* + *Sound level* + *Technical difficulty ratings* + *Expressive difficulty ratings* + (1|piece) + (1|ID) + ar1(bars + 0|piece:ID)
The formulation of Model 2 was as follows:
*Pupil size* ~ *Cloud diameter* + *Harmonic complexity ratings* + *Arm motion* + *Sound level* + *Technical difficulty ratings* + *Expressive difficulty ratings* + (1|piece) + (1|ID) + ar1(bars + 0|piece:ID)

To estimate effect sizes, we used a hierarchical modelling procedure in which significant predictors from Models 1 and 2 were added incrementally one by one to a null model containing only the intercept term and random effects. Predictors were entered in decreasing order of absolute estimate size (i.e., in the order they are listed in Table 1; see Data Sheet 1 in Supplementary Material). These hierarchically constructed models were then compared against the null model. We report χ^2^ tests and Bayesian Information Criterion (BIC) values as indications of effect size. BIC is commonly used as a criterion for model selection. To protect against overfitting, it incorporates a penalty for the number of predictors that are included in a model. Lower BIC indicates better support for a given model.

**Table 1:** Results of linear mixed effects modelling for performers.

**Model**	**Fixed effect**	**Estimate**	**SE**	***z*-value**	**χ^2^**	**BIC**
Model 1	(null model)	—	—	—	—	–1240.7
	Technical difficulty	0.0316	0.0038	8.21^***^	102.37^***^	–1334.8
	Cloud diameter	–0.0192	0.0029	6.56^***^	51.74^***^	–1378.4
	Harmonic complexity	–0.0120	0.0041	2.95^**^	6.88^**^	–1377.0
	Expressive difficulty	0.0098	0.0039	2.51^*^	5.42^*^	–1374.2
	QoM head	0.0003	0.0003	1.24	—	—
	Sound level	1.4e-5	1.4e-5	1.05	—	—
Model 2	(null model)	—	—	—	—	–907.40
	Technical difficulty	0.0216	0.0045	4.80^***^	33.53^***^	–933.10
	Harmonic complexity	–0.0188	0.0049	3.81^***^	21.22^***^	–946.48
	Cloud diameter	–0.0159	0.0033	4.80^***^	19.63^***^	–958.28
	QoM arms	0.0009	0.0001	6.49^***^	43.68^***^	–994.12
	Expressive difficulty	0.0057	0.0046	1.25	—	—
	Sound level	2.2e-5	1.59e-5	1.39	—	—

*Predictors are listed in descending order of absolute estimate size. Negative estimates indicate predictors that had a negative effect on pupil diameter. SE indicates standard error*.

* *p < 0.05*,

** *p < 0.01*,

*** *p < 0.001*.

#### 2.4.3. Effects of Rehearsal vs. Concert Setting, Musical Role, and Piece/Concert Time

To test for differences in levels of baseline arousal between the start of the rehearsal and the start of the concert, we compared the pupil diameters that were recorded in the first rehearsal baseline with the pupil diameters that were recorded in the baseline before the concert performance, using a Wilcoxon Signed Rank test.

We also compared average pupil diameters between the four concert pieces for each performer individually. Only data from the concert were used in this part of the analysis. Series of LMMs were run for each performer individually that included concert piece as a fixed effect. Concert piece was also included as a random effect in each model, and we specified an autocorrelation structure (order = 1) with time in concert piece as a covariate. We ran three models per performer with different concert pieces set as the base level for contrasts, so that we could get the full set of between-piece contrasts (6 total). We tested for significance at α =.008, following Bonferroni adjustment. Importantly, the “effect of concert piece” that we tested with these models reflects not only differences in musical material, but also the passing of concert time, and in some cases changes in musical role (the violinists exchanged places after Haydn II).

### 2.5. Results

#### 2.5.1. Linear Mixed Effects Modelling of Pupil Diameter

The reader is referred to Figure 1 for a reminder of which predictors we tested. The results of the LMMs are given in Table 1. Model 1 showed positive effects of Technical difficulty and Expressive difficulty on pupil size, and negative effects of Cloud diameter and Harmonic complexity. Head motion and Sound level did not yield significant effects. Models containing the four significant predictors were compared against a null model, and all predictors yielded significant χ^2^ values. However, only the model containing Technical difficulty and the model containing Technical difficulty and Cloud diameter improved the BIC, suggesting that the effects of Harmonic complexity and Expressive difficulty on pupil diameter were weak.

Model 2 showed positive effects of Arm motion and Technical difficulty on pupil size, and negative effects of Cloud diameter and Harmonic complexity. Sound level and Expressive difficulty did not yield significant effects. When we compared a null model against a hierarchical series of models containing the four significant predictors, all predictors yielded significant χ^2^ values and reduced the BIC relative to the null model.

In summary, both models showed stimulating effects of bodily effort (Technical difficulty, Arm motion) and negative effects of musical complexity (Harmonic complexity, Cloud diameter). Only Model 1 showed a stimulating effect of Expressive difficulty. Descriptive plots for significant predictors are given in the Supplementary Figures 1–5.

We also evaluated the similarity between predictors that could be expected to overlap. Table 2 lists the correlations between performers' rating measures. Table 3 lists the correlations between measures relating to bodily effort. The correlation between musical complexity measures (Harmonic complexity ratings and Cloud diameter) was slight, *r* =.28, *p* < .001.

**Table 2:** Correlations between performers' rating measures.

	**Harmonic complexity**	**Technical difficulty**
Technical difficulty	0.43^*^	—
Expressive difficulty	0.41^*^	0.58^*^

* *p < 0.001*.

**Table 3:** Correlations between measures relating to bodily effort.

	**Technical difficulty ratings**	**Sound level**
Head motion	–0.001	0.31^*^
Arm motion	0.02	0.41^*^
Sound level	0.08^*^	—

* *p < 0.001*.

#### 2.5.2. Effects of Rehearsal vs. Concert Setting, Musical Role, and Piece/Concert Time

**Figure 3:**
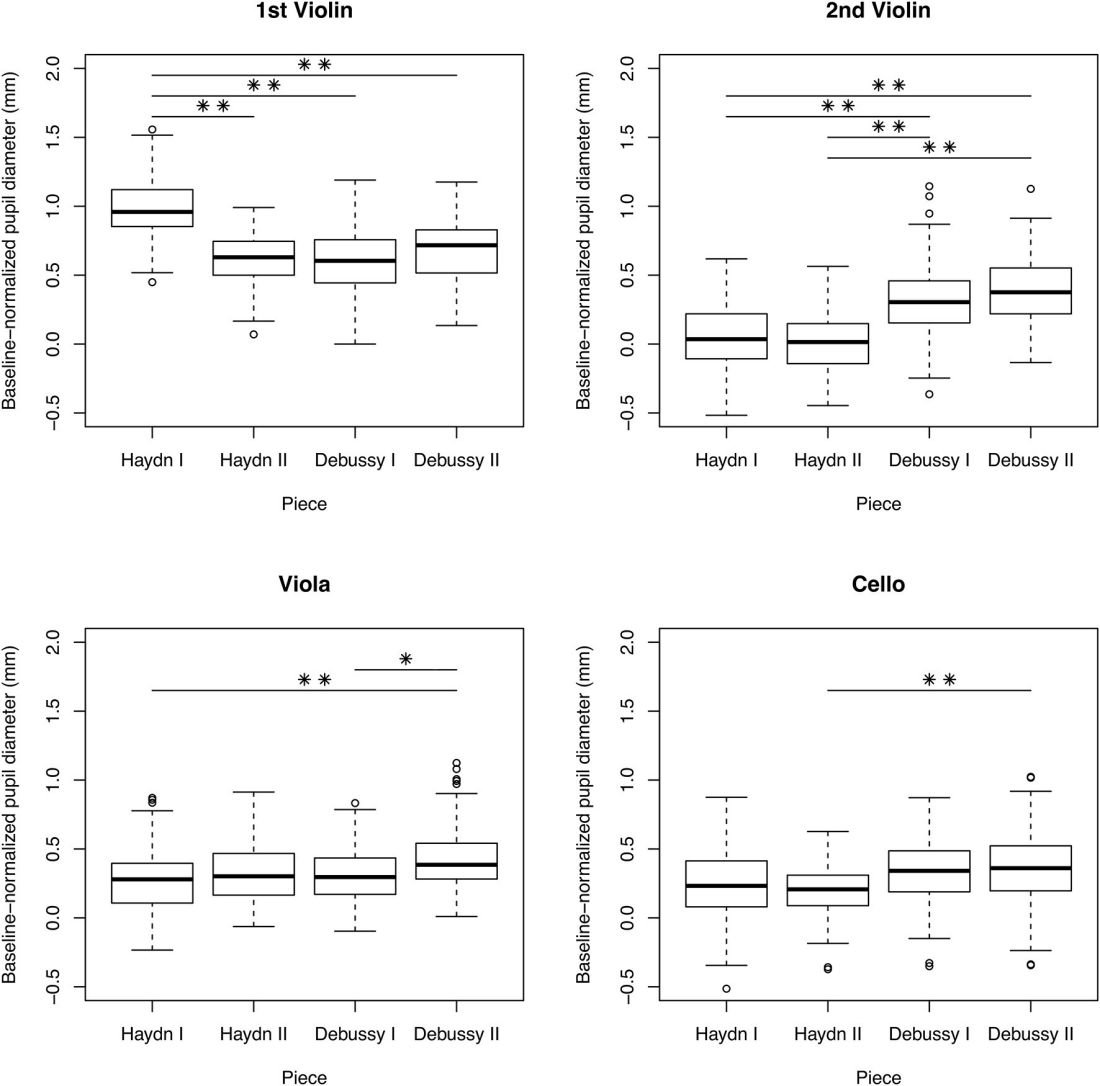
Boxplots showing pupil diameters across concert performances for each musician. Note that the violinists switched places for the Debussy pieces, so the 1st Violin played the 2nd Violin part and vice versa. **p < 0.001, *p < 0.008.

Figure 3 shows mean pupil diameters for each performer/piece combination. Our comparison of baseline pupil size captured before the first rehearsal with baseline pupil size captured before the first concert performances showed no significant difference, *M* = 3.72 mm, *SD* = 1.05 mm (rehearsal); *M* = 4.08 mm, *SD* =.96 mm (concert); *W* = 4, *p* =.34.

Results of the LMMs testing within-performer/between-piece difference in pupil diameter are shown in Table 4. For the 1st violinist, pupil dilation was greatest in the first piece (Haydn I). For the 2nd violinist, pupil dilation was greater in the Debussy pieces than in the Haydn pieces. This is notable because the 2nd violinist played as 1st violinist for the Debussy pieces. The violist showed greater pupil dilation in Debussy II than in Haydn I or Debussy I. The cellist showed greater pupil dilation in Debussy II than in Haydn II.

**Table 4:** Results of the LMMs testing within-performer/between-piece differences in pupil diameter.

**Performer**	**Contrast**	**Estimate**	**SE**	***z*-value**
1st Violin	Haydn I vs. Haydn II	–0.3331	0.0566	5.89^**^
	Haydn I vs. Debussy I	–0.3781	0.0494	7.65^**^
	Haydn I vs. Debussy II	–0.3327	0.0499	6.67^**^
	Haydn II vs. Debussy I	–0.0450	0.0564	0.80
	Haydn II vs. Debussy II	0.0004	0.0571	1.00
	Debussy I vs. Debussy II	0.0453	0.0499	0.36
2nd Violin	Haydn I vs. Haydn II	–0.0065	0.0556	0.012
	Haydn I vs. Debussy I	0.2653	0.0469	5.66^**^
	Haydn I vs. Debussy II	0.3363	0.0475	7.08^**^
	Haydn II vs. Debussy I	0.2718	0.0554	4.90^**^
	Haydn II vs. Debussy II	0.3428	0.0560	6.13^**^
	Debussy I vs. Debussy II	0.0710	0.0474	1.50
Viola	Haydn I vs. Haydn II	0.1322	0.0541	2.44
	Haydn I vs. Debussy I	0.0335	0.0452	.74
	Haydn I vs. Debussy II	0.1547	0.0458	3.38^**^
	Haydn II vs. Debussy I	–0.0987	0.0537	1.84
	Haydn II vs. Debussy II	0.0225	0.0539	0.42
	Debussy I vs. Debussy II	0.1212	0.0456	2.66^*^
Cello	Haydn I vs. Haydn II	–0.0460	0.0505	0.91
	Haydn I vs. Debussy I	0.0828	0.0417	1.99
	Haydn I vs. Debussy II	0.1110	0.0423	2.63
	Haydn II vs. Debussy I	0.1288	0.0505	2.55
	Haydn II vs. Debussy II	0.1569	0.0509	3.08^*^
	Debussy I vs. Debussy II	0.0281	0.0421	0.67

** *p < 0.001*,

* *p < 0.008*.

**Figure 4:**
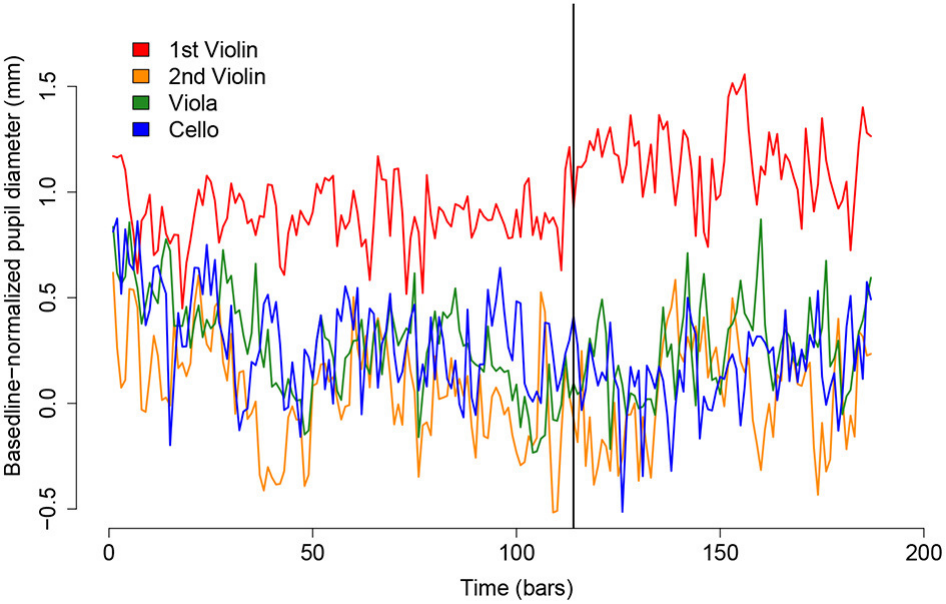
Timecourse of pupil diameters for the quartet across the course of the 1st movement of the Haydn String Quartet. The black vertical line indicates the location of the 1st violinist's failed page turn.

During the quartet's concert performance of Haydn I, an unexpected event occurred: the 1st violinist mishandled a page turn and, as a result, had to play the last page from memory. The sudden uptake in arousal and prolonged increase in mental effort are clear in the timecourse of his pupil diameter curve (Figure 4). We would note that this incident does not entirely account for the 1st Violinist's high average pupil diameter during that performance. As can be seen from the plot, the 1st Violinist's pupil was dilated (relative to the other musicians) from the start. We would also note that the 1st violinist's response to the page turn incident had no noticeable effect on the results that are presented in section 2.5.1. Models 1 and 2 were rerun on a data subset that excluded the 1st violinist after the moment of the error and the pattern of significant and nonsignificant effects remained the same.

### 2.6. Discussion

This experiment evaluated the contributions of musical complexity, bodily effort, and expressive difficulty to pupil size in performing musicians. In this section, we will focus on the effects of bodily effort, musical complexity, and expressive difficulty, which informed our decision to carry out Experiment 2. We will also discuss the effects of situational factors, including within-performer/between-piece differences in pupil size.

#### 2.6.1. Effects of Bodily Effort, Musical Complexity, and Expressive Difficulty on Pupil Size

Both Models 1 and 2 showed negative effects of Harmonic complexity and Cloud diameter on pupil size. We included Cloud diameter in our analysis as a more systematic measure of harmonic complexity to complement performers' subjective ratings. As we explained in the Introduction, Cloud diameter provides an indication of the degree of dissonance in each chord. It is notable that Cloud diameter and ratings of Harmonic complexity were only slightly correlated. The performers may have accounted for other aspects of harmonic complexity in their ratings (e.g., number of distinct tones, or amount of chord-to-chord change). Performers might also have weighted the relative complexity of chords within each bar less systematically than we achieved by calculating per-bar Cloud diameters. Despite the limited overlap between Cloud diameter and Harmonic complexity ratings, both measures yielded similar, unexpectedly negative effects on pupil size.

We had predicted that increased musical complexity would demand more effortful music processing and prompt increased pupil dilation, so this result was unexpected. Harmonic complexity is one component of the broader construct of musical complexity, and may share a complex relationship with other components, such as metric or rhythmic complexity, which also place (potentially competing) demands on attention. Thus, a potentially stimulating effect of Harmonic complexity might have been masked by other musical factors.

Harmonic complexity might also be less relevant to the experience of mental effort in string quartet performance than we originally predicted. Most quartets have spent a lot of time rehearsing by the time they perform in concert, and therefore have a close familiarity with the music. This familiarity might change the way they process the harmonic information that is contained in the music, perhaps reducing the mental effort that processing requires. In order to determine whether harmonic complexity affects performers and listeners differently, we designed Experiment 2 especially with the aim of examining the relationship between harmonic complexity and mental effort in listeners.

Performers' ratings of Technical difficulty had a positive effect on pupil size for both models. Indeed, for both models, Technical difficulty yielded a larger absolute estimate size than the other significant effects. These results are in line with our prediction that increased technical difficulty would engage increased mental effort. The Haydn and Debussy String Quartets are stylistically different and present performers with a variety of challenges. The performers used a large range of technical difficulty ratings for all pieces (1–6 for Haydn I, 1–5 for Haydn II, 1–7 for Debussy I, 1–6 for Debussy II), suggesting that they considered these variety of challenges in their evaluations. It is interesting that perceived technical difficulty seems to have a continued effect on mental effort when the musicians are playing well-practiced music and likely have many aspects of their bodily performance automatized.

Model 1 showed no effect of Head motion, while Model 2 showed a positive effect of Arm motion. For string musicians, head motion is primarily expressive. This is in contrast to arm motion, which is more directly involved in sound production. Arm motion happens at a much faster pace and must be controlled at a much finer level than is the case for head motion. In particular, control is needed for carrying out correct fingering and positioning the left hand so as to maintain intonation, as well as for carrying out appropriate bowing technique (Dalmazzo and Ramírez, 2019; D'Amato et al., 2020) and achieving coordination between the two hands. As a result, arm motion likely requires more substantial bodily effort than does head motion, and may have a more arousing effect on the body because it requires more physical exertion.

Model 1 showed a positive effect of Expressive difficulty on pupil size, in line with our prediction, though the effect was weak, and was not significant in Model 2. We presume that expressive difficulty incorporates a combination of demands relating to technical difficulty, complexity, emotional engagement, and perhaps coordination difficulty (which we did not evaluate here; see General Discussion). The positive effect that we observed in Model 1 suggests that this higher-order measure explains some variance in pupil diameter, above and beyond that explained by the other predictors relating to bodily effort and complexity.

In summary, both models showed positive effects of technical difficulty ratings and negative effects of Harmonic complexity ratings and Cloud diameter. The models differed in their results for body motion (quantity of arm motion had a positive effect on pupil size; quantity of head motion was nonsignificant) and expressive difficulty. As we have discussed, the difference in effects of head and arm motion likely has to do with the different musical functions that these parts of the body have, as well as the degree of exertion and control that they require. The effect of expressive difficulty in Model 1 was significant but weak. The lack of effect of expressive difficulty in Model 2 might be due to inclusion of arm motion instead of head motion. Perhaps arm motion overlaps with expressive difficulty to a greater extent than does head motion. Model 2 also used a reduced dataset due to the poor tracking of some arm markers, which may have rendered the already-weak effect of expressive difficulty less clear.

#### 2.6.2. Effects of Situational Factors on Pupil Size

While performers demonstrated a slightly larger pupil diameter at the start of the concert than at the start of the rehearsal, this difference was not significant. This lack of effect is in contrast to our prediction that arousal would be higher before the concert. The quartet may have been anxious at the start of the rehearsal, since they had not performed in our lab before, and had to get used to the unusual setting, the motion capture and eye tracking equipment, and the non-optimal acoustics. Therefore, they may have experienced a high baseline arousal at the very beginning of the session, which reduced as they acclimatized to the lab environment.

The lack of effect here reminds us that performance always occurs in the context of some social and material environment (van der Schyff et al., 2018), which unavoidably has some effect on performers' arousal. We should also be wary of blindly categorizing concert and rehearsal performances as high and low arousal. Performers may feel more pressure to perform well under some rehearsal conditions (e.g., when playing in an unfamiliar place, when rehearsing for the last time before an important concert) than in some concert conditions (especially if the concert is relatively low stakes). In future research, studies of arousal during public performance in ecological settings should take into account the performance environment and performers' goals and mindset, to show more clearly how changes in arousal relate to the performers' placement in a specific concert situation. This could be done with a mixed-methods approach that includes physiological/behavioural measures and interviews/questionnaires, similar to the paradigm proposed by Bojner Horwitz et al. (2020).

Our within-subject/between-piece analysis yielded some notable findings. The first violinist exhibited a very dilated pupil during the first piece (Haydn I). Based on our debriefing discussions with the musicians, we understand that he was feeling anxious at the start of the concert. This anxiety was exacerbated by a failed page turn partway through Haydn I, which necessitated him to play the last pages of the piece by memory. Although his pupils remained dilated through the rest of the concert relative to the other quartet members, we did see a significant reduction in his pupil size between Haydn I and Haydn II. The second violinist showed smaller pupil sizes during the Haydn pieces, when he was playing as second violin, than during the Debussy pieces, when he was playing as first violin. Thus, both violinists showed greater arousal when playing as first violin than when playing as second violin. This is in line with the prediction that the first violin leadership role comes with additional demands (Davidson and Good, 2002; Timmers et al., 2014; Glowinski et al., 2015). As we have previously reported, during the rehearsal performances, the first violinist was distinct in his visual attention, and almost never looked at any of the other musicians^2^. In contrast, the other musicians looked at him more than they looked at any other quartet member. Combined with the current results, it seems that the whole quartet recognized the first violinist as the leader, and that this had substantial effects on how everyone interacted with each other.

## 3. Experiment 2: Mental Effort in Music Listening

Experiment 2 was designed to follow up on some of the findings from Experiment 1. Given the small sample size in Experiment 1 (n = 4), we wanted to test whether the effects that we observed there would reemerge in a larger sample. A follow-up experiment with listeners would also allow us to shed some light on how mental effort and arousal compare across performance and listening tasks. An especially interesting question is how much performers' bodily effort contributes to the experiences of musically-trained listeners. Does technical difficulty also demand increased mental effort among listeners? Another interesting question is how strongly performers' subjective ratings of difficulty and complexity contribute to listeners' experiences. While we would expect some widespread agreement on what is complex or difficult, performers differ in their skills and anatomical/physiological constraints (e.g., hand size, strength, ability to move rapidly, etc.), so they necessarily show some variability when rating these factors. If the subjective ratings given by a small sample of four performers contribute significantly to listeners' mental effort, then this will indicate some generalizability to those ratings.

### 3.1. Participants

Sixteen trained musicians (8 female/8 male) completed the listening task. Five of the musicians were violinists (3), violists (1), or cellists (1), and rest of the musicians played a variety of other instruments (guitar–3, piano–3, flute–1, percussion–1, sitar–1, trombone–1, voice–1). Separate analyses were run for string and non-string musicians, but revealed no between-group differences, so we merged all participants together into a single group. The musicians were on average 26.4 years old (*SD* = 5.7) and had on average 13.3 years of musical training (*SD* = 6.7).

We asked the musicians to rank their familiarity with each piece on a scale of 1-4 (1 = “Never heard it before"; 2 = “I think I've heard it before"; 3 = “I've heard it before"; 4 = “I've played it before"). Scores averaged across listeners indicated low familiarity with the music (1.94 and 2.00 for Haydn movements I and II; 1.88 and 1.56 for Debussy movements I and II).

### 3.2. Materials and Equipment

Listeners heard the music from Creative A50 speakers, adjusted to a comfortable volume, while pupil data was collected from a stationary eye tracker (SMI iView RED) at 60 Hz. Listeners rested their chin and forehead on a chinrest positioned 70 cm from a computer screen. The experiment was run through SMI Experiment Center, which collected pupil data and presented audio/visual stimuli. During listening trials, the screen featured a white background with a black outline of a circle. Listeners were instructed to keep their eyes fixated within the circle.

### 3.3. Procedure

Participants listened to the recordings in the same order that they were performed (Haydn 1st movement, Haydn 2nd movement, Debussy 1st movement, Debussy 2nd movement). They were given the name and composer of each piece and asked to keep their eyes open and fixated on the computer screen while listening. A 60-s baseline pupil measurement was taken prior to each listening trial. Following each trial, the participants were asked to rate their familiarity with the piece they had just heard, and then allowed to take a break before continuing. Following the final listening trial, they answered some questions about their musical background.

### 3.4. Analysis

#### 3.4.1. Preprocessing of Pupil Data

We used the same preprocessing procedure as in Experiment 1 (Section 2.4.1).

#### 3.4.2. Linear Mixed Effects Modelling of Pupil Diameter

We used the same modelling procedure as in Experiment 1. For ratings of Technical difficulty, Harmonic complexity, and Expressive difficulty, we averaged the performers' ratings at each bar to get a single series of values per predictor. Similarly, for Head and Arm, we averaged quantity of motion values across performers at each bar. Effect sizes for significant predictors were estimated using the same hierarchically modelling procedure as in Experiment 1.

### 3.5. Results

The results of the LMM are given in Table 5. Both models showed a positive effect of Expressive difficulty and negative effects of Harmonic complexity and Technical difficulty on pupil diameter. Model 1 showed a negative effect of Head motion, and Model 2 showed a negative effect of Arm motion. Cloud diameter and Sound level did not yield significant effects for either model. The four significant predictors also showed significant χ^2^ values when added to a null model and reduced the BIC (although the reduction by technical difficulty was very small). Descriptive plots for significant predictors are given in the Supplementary Figures 1–3,5,6.

**Table 5:** Results of linear mixed effects modelling for listeners.

**Model**	**Fixed effect**	**Estimate**	**SE**	***z*-value**	**χ^2^**	**BIC**
Model 1	(null model)	—	—	—	—	–3164.9
	Harmonic complexity	–0.0426	0.0040	10.60^***^	126.00^***^	–3281.7
	Expressive difficulty	0.0347	0.0041	8.52^***^	98.94^***^	–3371.4
	Technical difficulty	–0.0100	0.0002	2.50^*^	9.64^**^	–3371.9
	QoM head	–0.0023	0.0002	10.31^***^	103.47^***^	–3466.1
	Cloud diameter	–0.0008	0.0017	0.45	—	—
	Sound level	1.4e-5	9.3e-6	1.53	—	—
Model 2	(null model)	—	—	—	—	–3164.9
	Harmonic complexity	–0.0456	0.0040	11.36^***^	126.00^***^	–3281.7
	Expressive difficulty	0.0372	0.0041	9.13^***^	98.94^***^	–3371.4
	Technical difficulty	–0.0101	0.0040	2.51^*^	9.64^**^	–3371.9
	QoM arms	–0.0006	9.7e-5	6.20^***^	37.95^***^	–3400.6
	Cloud diameter	–9.7e-5	0.0017	0.06	—	—
	Sound level	6.8e-6	0.0040	0.73	—	—

* *p < 0.05*,

** *p < 0.01, and*

*** *p < 0.001*.

### 3.6. Discussion

This experiment tested the effects of musical complexity, performers' bodily effort, and performers' ratings of expressive difficulty on pupil size in musically-trained listeners. In line with Experiment 1, we observed negative effects of musical complexity and a positive effect of expressive difficulty. In contrast to Experiment 1, this experiment showed significant negative effects of quantity of head and arm motion and performers' ratings of technical difficulty on listeners' pupil sizes. This result was not in line with our prediction that “traces” of performers' bodily effort would “sound” through the music and demand increased mental effort and arousal during listening.

In the literature, there is convergent evidence from many brain imaging and behavioural studies that music listening engages motor circuits (Novembre and Keller, 2014). However, questions remain regarding the extent to which this motor engagement is necessarily an active, attention-drawing process. We presented our participants with a passive listening task about 20 min in duration. Though all of our participants were trained musicians, their musical background and interests varied and several of them were not familiar with string quartet repertoire. Thus, some of our participants may not have been very actively engaged in listening. Furthermore, to maximize quality of pupil data, we did not include video in the stimulus presentation. Visual presentation of performers' gestures can activate covert simulation mechanisms in observers, and might have encouraged motor engagement from our participants (Haslinger et al., 2005; Wöllner and Cañal-Bruland, 2010; Su and Pöppel, 2012; Taylor and Witt, 2014).

We observed a negative effect of performers' ratings of Harmonic complexity on pupil size. The effect of Cloud diameter was also negative, but nonsignificant. This finding is in line with the results of Experiment 1, and in conflict with our original prediction that harmonic complexity would have a stimulating effect on mental effort. We propose an explanation for these findings in the General Discussion.

Finally, we observed a positive effect of expressive difficulty for listeners. This is in line with our prediction, though in contrast to our findings in Experiment 1, where expressive difficulty showed no effect. The larger sample size in Experiment 2 may have allowed this effect to come through. Expressive difficulty or intensity may also account for more of the variance in pupil size during music listening than during music performance, since there are fewer demands on the motor system. We explore this effect in greater depth in the General Discussion.

## 4. General Discussion

This study investigated the effects of harmonic complexity, bodily effort, and expressive difficulty on mental effort and arousal during music performance and listening. Pupil diameter was used to estimate mental effort and arousal. In Experiment 1, we collected pupil data from the members of a student string quartet as they performed in rehearsal and concert settings in our lab. In Experiment 2, we collected pupil data from a sample of trained musicians as they listened to the quartet's concert recordings.

Our results revealed stimulating effects of bodily effort and expressive difficulty on performers' pupil size, and stimulating effects of expressive difficulty on listeners' pupil size, in line with our predictions. Contrary to our predictions, we also observed consistently negative effects of Harmonic complexity and Cloud diameter (i.e., harmonic dissonance) for both performers and listeners, and negative effects of performers' bodily effort on listeners. Finally, we saw elevated levels of arousal in both violinists during their turns as 1st violinist. These findings are discussed in more detail below.

### 4.1. Body Motion, Technical Difficulty, and Sound Level

Performers' arm motion and perceived technical difficulty had stimulating effects on their pupil diameter. These effects supported our prediction that bodily effort would contribute to mental effort and arousal. Our results showed that arm motion improved the fit of a model that already included technical difficulty, suggesting that it accounted for a unique part of the variance. These findings are in line with previous studies of musical effort, which show greater pupil dilation in overt performance than in listening or imagined performance, suggesting an increased demand on cognitive resources (O'Shea and Moran, 2019; Endestad et al., 2020). A remaining question is whether we can identify unique effects of motor exertion and the mental effort involved in motor control on pupil size. The design of the current study did not enable us to make this distinction; however, future studies might present performers with a task that independently varies exertion and complexity.

Our other two measures of bodily effort—quantity of sound level and head motion—did not yield significant effects. The lack of effect for sound level suggests that this is not a strong predictor of mental effort, despite the relationship between sound level and physical force. Head motion, for string players, is primarily expressive and does not generally require as much motor control or physical strength as does arm motion, which is directly involved in sound production (see Discussion 1). This could partially explain the lack of stimulating effect of head motion on pupil size.

Expressive non-sound-producing head or body motion might indeed require relatively little mental effort overall, especially for experienced musicians who are playing well-practiced repertoire, and might even facilitate structuring of the performance. Expressive body motion is an integral component of expressive performance (Glowinski et al., 2013b; Chang et al., 2019). Skilled musicians reduce their body motion substantially when asked to play deadpan, even if no specific instructions regarding body motion are given (Davidson, 2007; Thompson and Luck, 2012). When asked to given an “immobile” performance, however, some slight expressive motion persists (Wanderley, 2002; Wanderley et al., 2005). Thus, to some degree, expressive body motion may occur automatically as a result of the performer's embodied relationship with the music (van der Schyff et al., 2018; Høffding and Satne, 2019). Still, further research is needed to show under what conditions expressive body motion requires more mental effort. In particular, it would be interesting to test whether expressive body motion reduces when other aspects of performing increase in difficulty.

For listeners, performers' head and arm motion and ratings of technical difficulty had a negative effect on pupil size. As we explained in the Discussion of Experiment 2, the lack of positive effect might be attributable to participants adopting a passive listening style during the experiment. Listeners might also not have perceived such substantial variability in the technical demands that were presented by the Haydn and Debussy selections, or they might have perceived difficulty across longer timeframes than we accounted for. For example, perhaps we would see different responses to music with long alternating periods of greater and lesser technical difficulty. Our measures of performers' bodily effort might also co-vary with another aspect of musical structure that our analysis did not capture (e.g., changes in timbre or tone quality, or phrase structure), that had a stronger effect on mental effort, resulting in a seemingly negative relationship between bodily effort and pupil size. While it might be useful in this case to consider other variables relating to musical structure as possible co-predictors, with the modelling approach that we used, care must be taken to avoid overfitting. A more effective approach might be to compare pupil responses to stimuli that vary more strongly in their physical and technical demands.

### 4.2. Harmonic Complexity, Tonal Tension, and Expressivity

Both experiments revealed negative effects of harmonic complexity (measured using performers' ratings and Cloud diameter) on pupil size, in contrast to our predictions. Harmonic complexity is just one component of musical complexity. While it might substantially engage listeners' attention when all other components are constant, this does not happen in real music; instead, different structural components might vary simultaneously, forming combined demands on attention that fluctuate over time. Thus, the negative effect that we saw on pupil size may reflect the relationships that harmonic complexity shares with other (untested) components of musical structure.

We should also note that for repertoire in the Western classical music tradition prior to the early twentieth century, harmonic complexity varies within fairly strict bounds (although these bounds changed over time, and differ between the Haydn and Debussy selections that we studied). In some music, periods of extreme harmonic complexity (e.g., as we see in some contemporary classical music that makes use of atonality) might make heightened demands on attention that outweigh the demands made by other structural features. In such cases, we might see a clear relationship between harmonic complexity and pupil size, despite the fact that harmony is embedded in a larger musical structure (and despite the passive rather than active/analytical listening task). However, even the more varied harmony present in Debussy's music is unlikely to achieve these extremes. During periods of high complexity, we might alternatively expect to see effects of listeners “zoning out” (mind wandering) if following the music proves too difficult or effortful (Unsworth and Robison, 2017; O'Shea and Moran, 2019). This would cause a reduction in pupil size. However, we would expect this reduction to endure over a relatively long period of time, not fluctuate from bar to bar.

Overall, we interpret these results as suggesting that individual low-level components of musical structure may not provide useful predictions of mental effort for performers or listeners. Higher-order predictors, which represent combinations of structural components, may be more effective. Indeed, performers' ratings of expressive difficulty—a measure which we presume is informed by musical complexity, technical difficulty, and emotional intensity—did predict pupil size for performers and listeners. The effect was slightly weaker for performers (significant only in Model 2), possibly because of conflicting demands by the motor system.

For ensemble players, an additional higher-order variable that might relate to pupil size is subjective ratings of coordination difficulty (how difficult is it for the ensemble to play together as a unit?). This variable would draw on factors relating to complexity, expressivity, and technical difficulty, as well as the relationships between parts in the ensemble (Keller et al., 2014)^1^. For classical ensembles playing well-practiced music, most of the major interpretive decisions have already been made, but coordination can still be challenging if the performers vary aspects of their practiced performance. Certain musical passages may continue to pose a challenge even after some practice; for example, long pauses followed by synchronized chords may continue to require effortful coordination (Bishop et al., 2019b). Future research could use pupillometry measures to investigate the relationship between coordination demands resulting from different structural contexts and mental effort.

The results of this study could be interpreted in terms of the adaptive-gain theory, which posits that exploitative and explorative control states underlie optimal performance on behavioural tasks (Aston-Jones and Cohen, 2011). These control states are mediated by the locus coeruleus-norepinephrine system. Exploitation is associated with phasic LC activity, intermediate pupil sizes, and increased responsivity to task-relevant stimuli, while exploration is associated with tonic LC activity, large pupil sizes, and facilitated processing of task-irrelevant stimuli or behaviour (Jepma and Nieuwenhuis, 2011). Musicians may switch between exploitative and explorative modes during performance as the musical demands change, resulting in fluctuations in pupil size.

### 4.3. Use of Mobile Eye Tracking in Music Performance Settings

This study is the first to use pupillometry to explore the relationships between cognitive-motor task demands and mental effort during ensemble performance in an ecological setting. Outside of a controlled lab environment, there are several factors that may add noise to pupil data. Three primary types of pupil response have been described in the literature: the pupil light response, which involves a pupil constriction in response to increased brightness; the pupil near response, which involves a pupil constriction when gaze shifts from a further-away object to a nearer object; and the psychosensory pupil response, which involves a pupil dilation in response to increased arousal or mental effort (Mathôt, 2018).

In our data collection, the performers unavoidably encountered changes in brightness and shifted their focus between nearer and further-away objects as their gaze moved between different parts of the visual scene. We attempted to minimize the effects of brightness by controlling the lighting of the performance space (i.e., using a constant, moderately bright level of room lighting instead of any stage lighting) and by covering the walls and windows with black curtains, to avoid any stray lights shining into the space and lighting contrasts between the white walls and dark floor. These controlled lighting conditions were also needed to minimize extraneous reflections for motion capture. The musicians played from scores, which allowed for some consistency in terms of brightness and distance in the visual display; however, they did not look exclusively at their scores throughout the performances. Ensemble musicians often spend some playing time watching their co-performers (Vandemoortele et al., 2018; Bishop et al., 2019a), and this was also the case in the current study (see results in^2^).

Of course, our attempts to control the performance space did have some effect on the musicians' experience, reducing the ecological validity of the performance. In particular, the musicians struggled with the dry acoustics of the space. In future studies of mental effort in music performance, we would recommend having musicians play from a score or fix their eyes on an empty screen or stand, although we would also note that instructing performers to restrict their gaze to the score can have some unintended effects on how they behave. Our analysis of the quartet's body motion showed lower quantity of motion in two rehearsal performances where they could look only at the score than in the other “free gaze” conditions see^2^.

The lack of control in Experiment 1 might have introduced noise into our pupil data. More concerning is the possibility of systematic effects. For example, the musicians might have looked away from the score during “easier” passages, and returned their gaze to the score during more “difficult” passages. In such cases, pupil constrictions (triggered by looking at the relatively bright and nearby score) might occur in association with increasing piece difficulty. Our results showed the opposite effect for technical and expressive difficulty, which stimulated pupil dilation rather than constriction. We did observe a negative relationship between pupil dilation and harmonic complexity measures; however, the same negative effect occurred for listeners in Experiment 2.

We did not attempt to compensate for effects of brightness or distance in our data analysis. We might have removed data segments where performers were not looking at the score, for example, but this would have reduced our dataset dramatically, and much more for some musicians than others. The musicians differed greatly in what percentage of performance time they spent looking at the score vs. at their co-performers. The cellist, in particular, sometimes spent as little as 40% of performance time looking at the score. Instead, we chose to complement our performance experiment with a listening experiment, which included a larger sample of remote participants, who completed their task in controlled conditions in the laboratory. With the listening experiment, we were able to confirm some of the results suggested by our performers' data as well as address some additional predictions.

As we showed in this study, overt body motion has an effect on pupil size. Pupillometry studies using music performance (or sports performance, etc.) paradigms have a unique opportunity to examine this relationship. In our case, we organized a recording set-up that would allow us to collect synchronized motion capture and eye tracking/pupillometry data. With motion capture data for performers heads and arms, we were able to show how these different types of movement have different effects. As we mentioned above, difficulties in distinguishing between the effects of body motion and the effects of other task demands on pupil size can also be problematic for researchers who want to measure these effects in isolation. In such cases, careful construction of an experimental paradigm that controls for different task demands would be needed.

Our ecological data capture gave us the unique opportunity to document a performance disruption: the 1st Violinist's missed page turn during the concert performance of the 1st movement of the Haydn String Quartet, which necessitated him to play the final page by memory. His pupils remained dilated throughout the rest of the piece, indicating heightened demands on mental effort. This type of performance disruption, along with the performer's natural reaction, would be hard to capture (or trigger) under more controlled, less ecological conditions. Our capture of this disruption shows how the violinist was able to successfully cope with the disruption by continuing to play from memory, without overt acknowledgment of the error. His individual coping response made the group resilient to greater disruption from the error (Glowinski et al., 2016).

### 4.4. Conclusions

This study shows an overlap in how performers and listeners attend to string quartet music from the Western classical repertoire. Both performers and listeners responded to changes in expressivity and musical structure, and performers' arousal levels were predicted by their sound-producing arm motion. The violinists in the quartet also both showed heightened arousal when performing as first violinist, suggesting that heightened stress may be associated with this role. Our study also demonstrates how mental effort and arousal can be successfully assessed using eye tracking and motion capture technologies in a relatively naturalistic concert setting. In the future, it would be valuable to build on our findings with studies of how coordination difficulty contributes to mental effort in ensemble performance, and to distinguish between the effects of overt motion and the mental effort associated with motor control on performers' pupil size.

## Data Availability Statement

The data for this study are openly available in the Quartet Body Motion and Pupillometry Database (10.5281/zenodo.4888176).

## Ethics Statement

This study was approved by the Norwegian Center for Research Data (NSD), with the project identification number 748915. Written informed consent was obtained from participants for the publication of potentially identifiable images and data.

## Author Contributions

LB, BL, and AJ conceived the experiment. LB collected the data, ran the analysis, and wrote the first draft of the paper. BL and AJ consulted on the analysis and contributed to the writing of the manuscript. All authors contributed to the article and approved the submitted version.

## Conflict of Interest

The authors declare that the research was conducted in the absence of any commercial or financial relationships that could be construed as a potential conflict of interest.

## Footnotes

^1^Bishop, L., and Keller, P. (forthcoming). “Instrumental ensembles,” in *The Oxford Handbook of Music Performance*, ed G. McPherson.

^2^Bishop, L., Gonzalez Sanchez, V., Laeng, B., Jensenius, A. R., and Høffding, S. (submitted). Variability of head motion and gaze across social contexts in string quartet performance.

^3^Both systems actually recorded the full experiment, and (Bishop and Jensenius, 2020) shows that they were comparable in recording quality. We used OptiTrack data for performance 7 because the Qualisys recording was started a few seconds late.

^4^kunstderfuge.com

^5^musescore.com

## Supplementary Material

Supplementary Figure 1 Violin plots showing the distribution of Quantity of Arm Motion values for each musician across pieces.

Supplementary Figure 2 Violin plots showing the distribution of ratings of Technical difficulty given by each musician across pieces.

Supplementary Figure 3 Violin plots showing the distribution of ratings of Harmonic Complexity given by each musician across pieces.

Supplementary Figure 4 Violin plots showing the distribution of Cloud diameter values across pieces.

Supplementary Figure 5 Violin plots showing the distribution of ratings of Expressive difficulty given by each musician across pieces.

Supplementary Figure 6 Violin plots showing the distribution of Quantity of Head Motion values for each musician across pieces.
