# Supplementary figures and images for: Musical and Bodily Predictors of Mental Effort in String Quartet Music: An Ecological Pupillometry Study of Performers and Listeners
Source: Front Psychol. 2021 Jun 28;12:653021. doi: 10.3389/fpsyg.2021.653021 (PMC8274478; doi:10.3389/fpsyg.2021.653021)

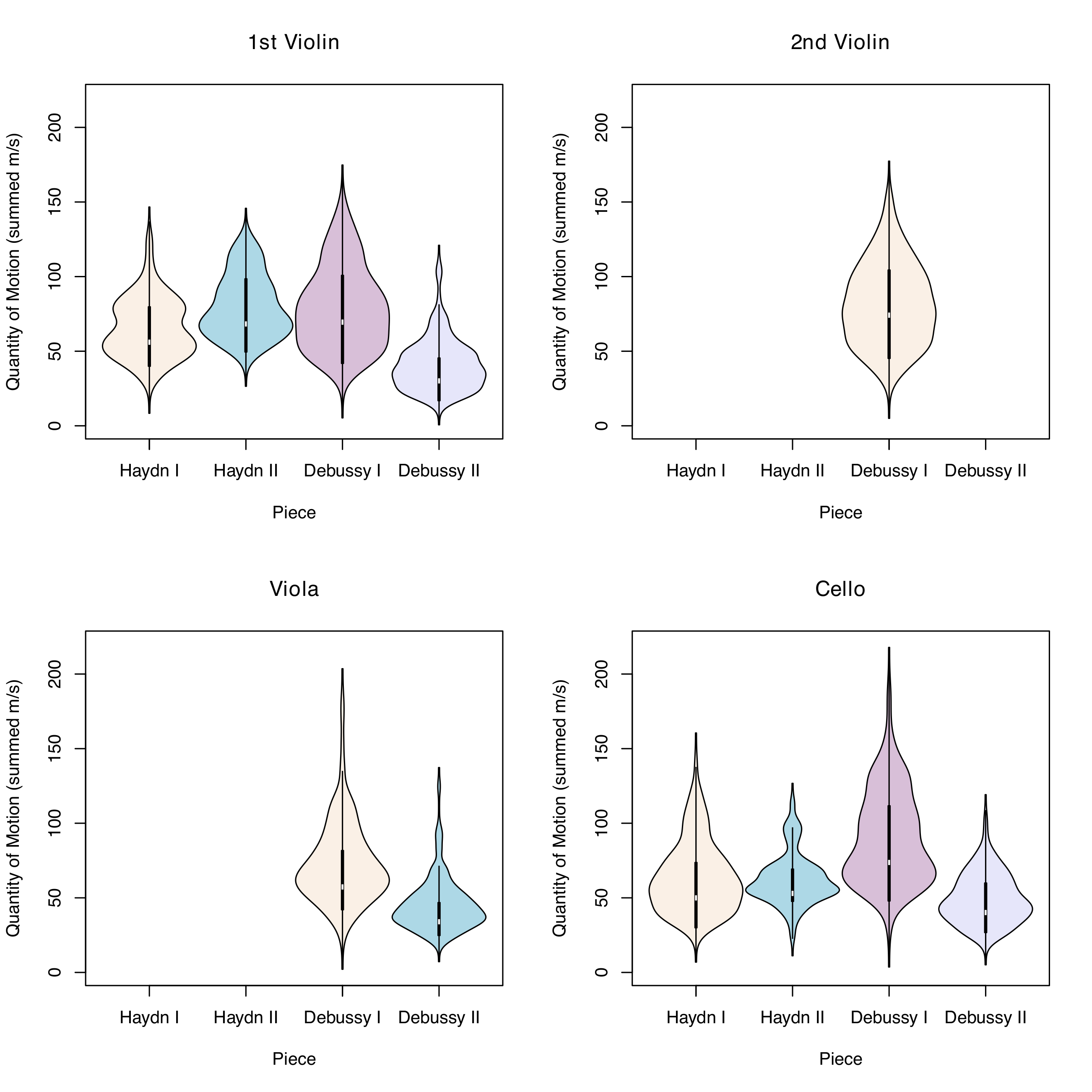

Supplement: Supplementary Figure 1 — Violin plots showing the distribution of Quantity of Arm Motion values for each musician across pieces. [file Image_1.tif]

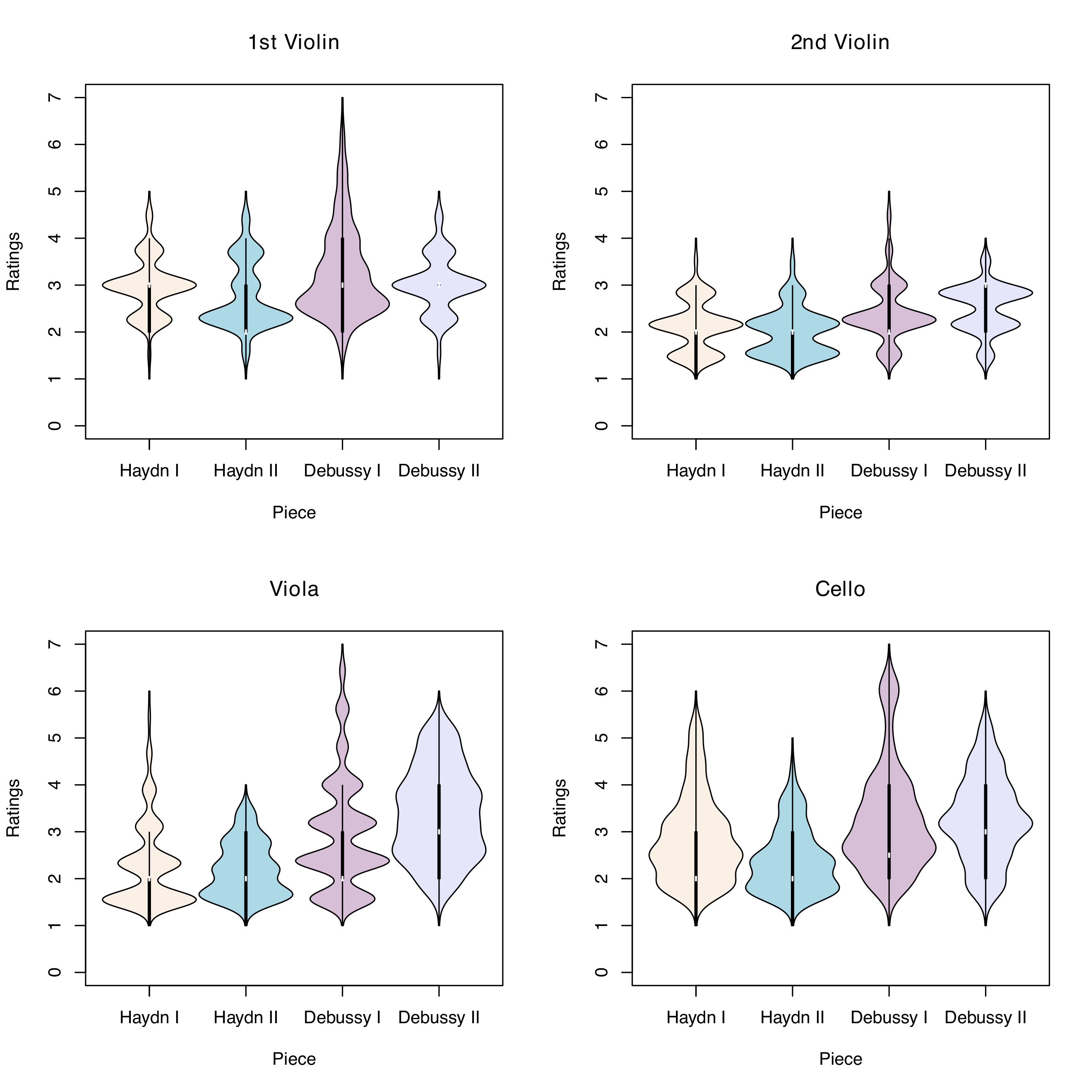

Supplement: Supplementary Figure 2 — Violin plots showing the distribution of ratings of Technical difficulty given by each musician across pieces. [file Image_2.tif]

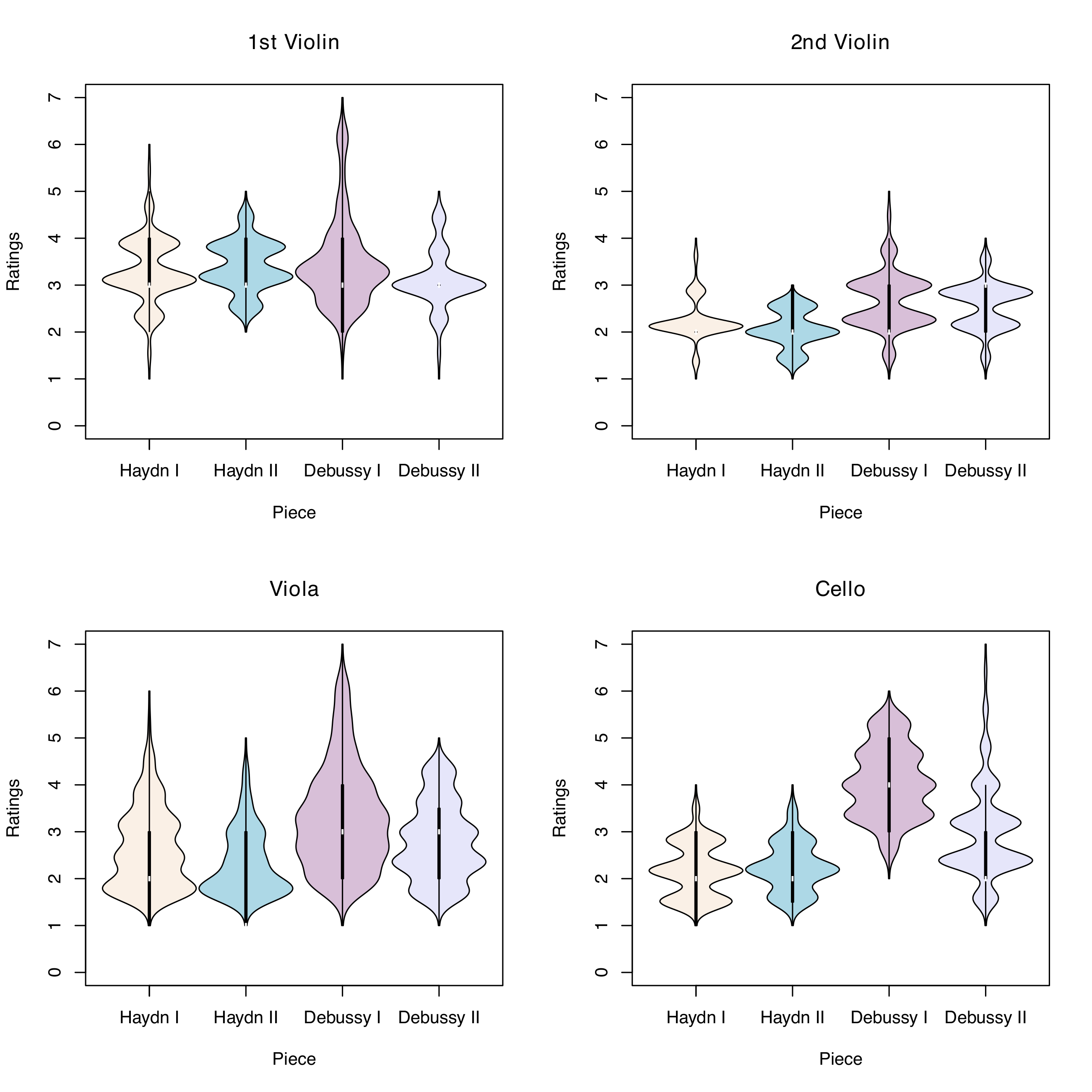

Supplement: Supplementary Figure 3 — Violin plots showing the distribution of ratings of Harmonic Complexity given by each musician across pieces. [file Image_3.tif]

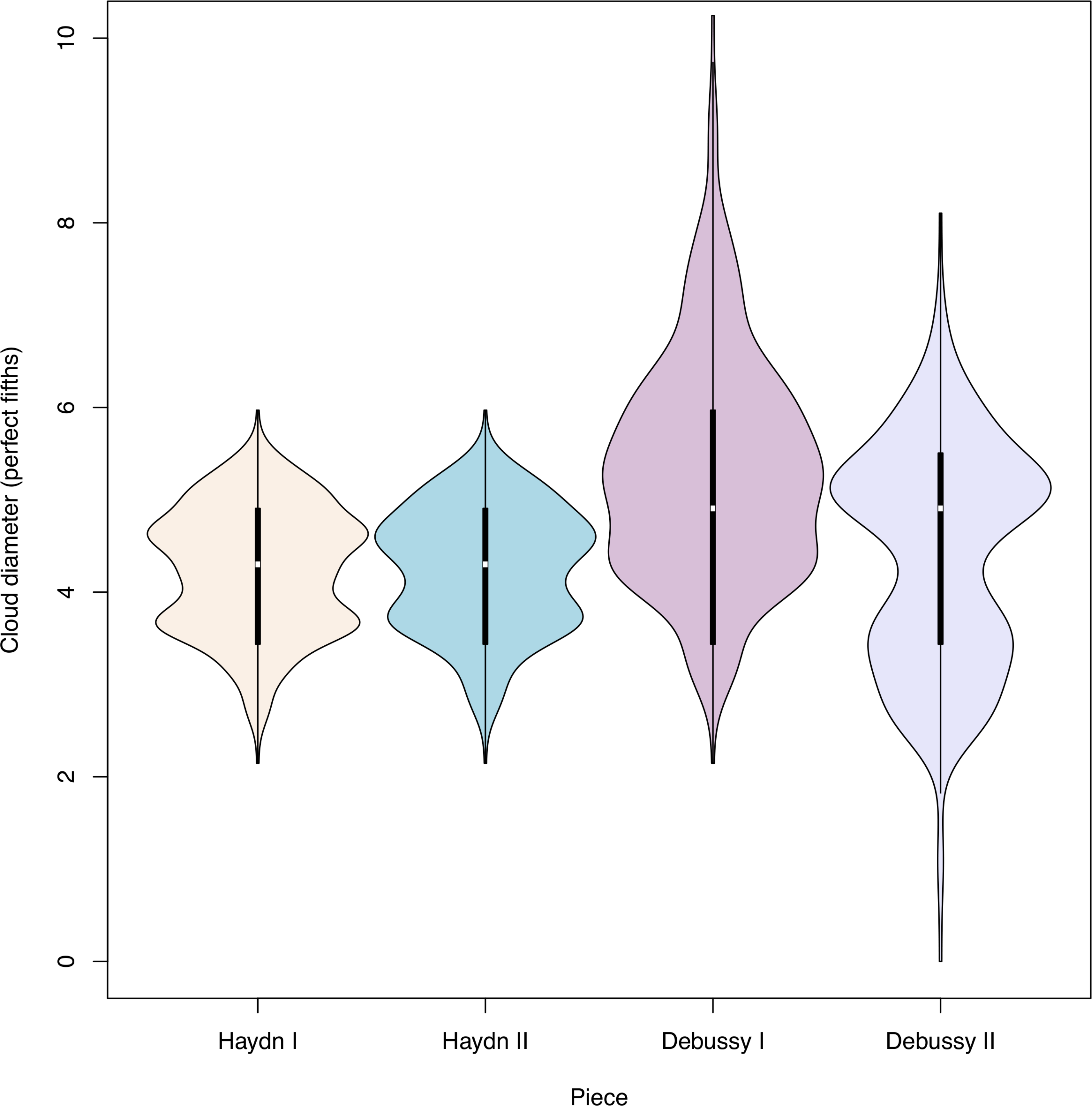

Supplement: Supplementary Figure 4 — Violin plots showing the distribution of Cloud diameter values across pieces. [file Image_4.tif]

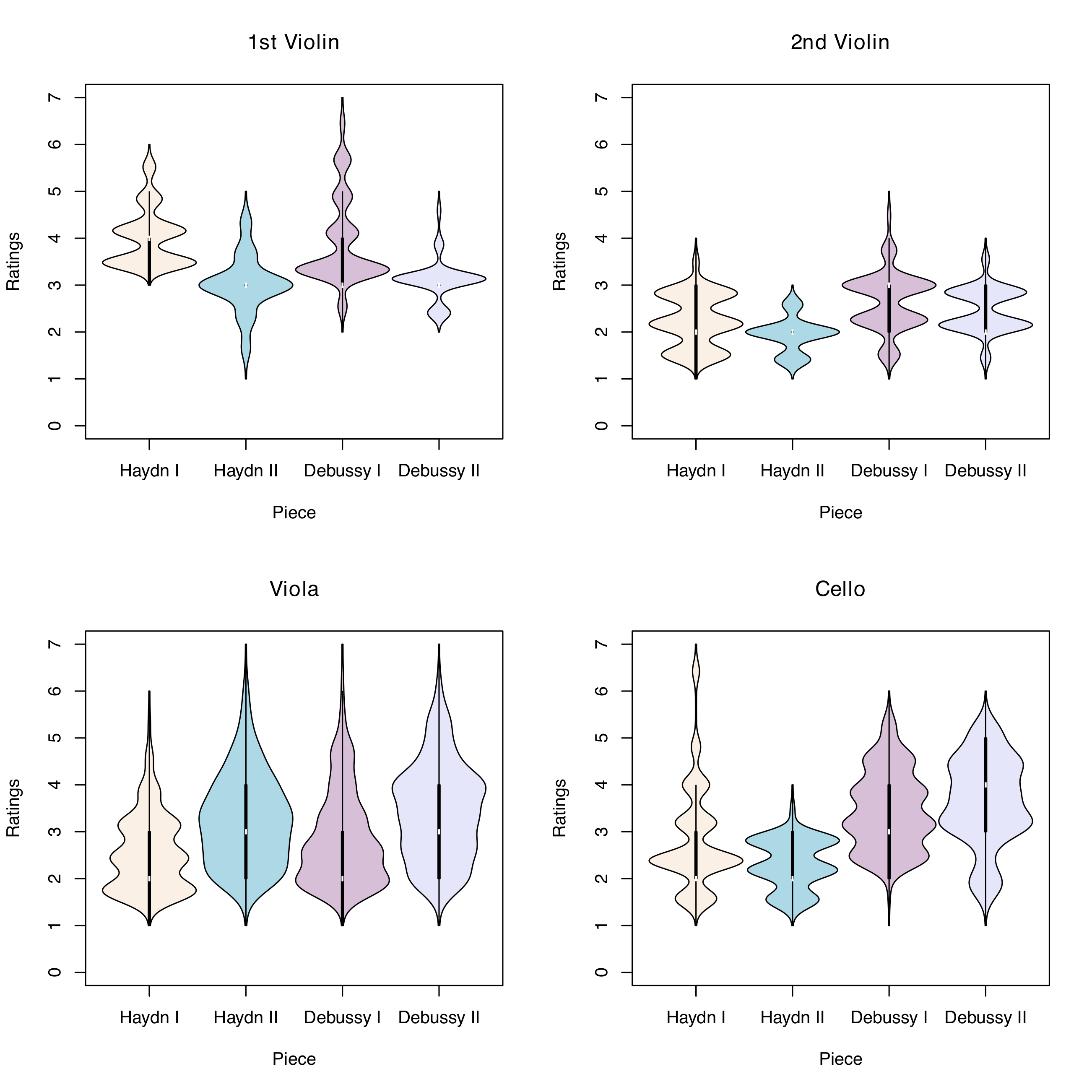

Supplement: Supplementary Figure 5 — Violin plots showing the distribution of ratings of Expressive difficulty given by each musician across pieces. [file Image_5.tif]

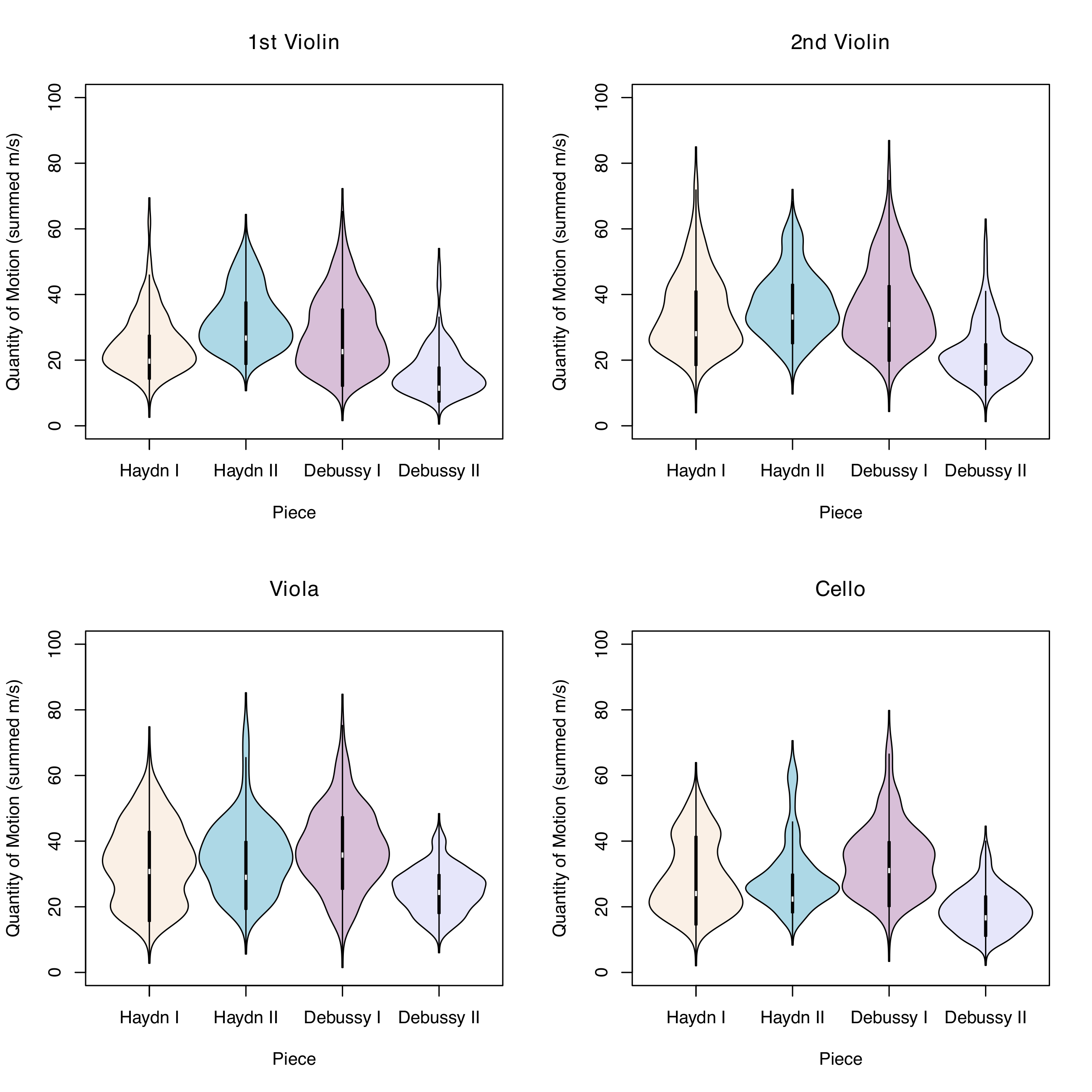

Supplement: Supplementary Figure 6 — Violin plots showing the distribution of Quantity of Head Motion values for each musician across pieces. [file Image_6.tif]
