# Supplementary material for: Musical and Bodily Predictors of Mental Effort in String Quartet Music: An Ecological Pupillometry Study of Performers and Listeners
Source: Front Psychol. 2021 Jun 28;12:653021. doi: 10.3389/fpsyg.2021.653021 (PMC8274478; doi:10.3389/fpsyg.2021.653021)
Supplement: Supplementary file 7 [file Data_Sheet_1.pdf]

Supplementary Material

Below are listed the formulations of the hierarchical models that were tested in Experiments 1 and 2 in order to assess effect sizes.

Experiment 1

Model 1 (including head motion)

(1) *Pupil size* ∼ *Technical difficulty ratings*
+ (1—piece) + (1—ID) + ar1(bars + 0—piece:ID)

(2) *Pupil size* ∼ *Technical difficulty ratings* + *Cloud diameter*
+ (1—piece) + (1—ID) + ar1(bars + 0—piece:ID)

(3) *Pupil size* ∼ *Technical difficulty ratings* + *Cloud diameter* + *Harmonic complexity ratings*
+ (1—piece) + (1—ID) + ar1(bars + 0—piece:ID)

(4) *Pupil size* ∼ *Technical difficulty ratings* + *Cloud diameter* + *Harmonic complexity ratings*
+ *Expressive difficulty ratings*
+ (1—piece) + (1—ID) + ar1(bars + 0—piece:ID)

Model 2 (including arm motion)

(1) *Pupil size* ∼ *Technical difficulty ratings*
+ (1—piece) + (1—ID) + ar1(bars + 0—piece:ID)

(2) *Pupil size* ∼ *Technical difficulty ratings* + *Harmonic complexity ratings*
+ (1—piece) + (1—ID) + ar1(bars + 0—piece:ID)

(3) *Pupil size* ∼ *Technical difficulty ratings* + *Harmonic complexity ratings* + *Cloud diameter*
+ (1—piece) + (1—ID) + ar1(bars + 0—piece:ID)

(4) *Pupil size* ∼ *Technical difficulty ratings* + *Harmonic complexity ratings* + *Cloud diameter*
+ *Quantity of Arm motion*
+ (1—piece) + (1—ID) + ar1(bars + 0—piece:ID)

Experiment 2

Model 1 (including head motion)

(1) *Pupil size* ∼ *Harmonic complexity ratings*
+ (1—piece) + (1—ID) + ar1(bars + 0—piece:ID)

(2) *Pupil size* ∼ *Harmonic complexity ratings* + *Expressive difficulty ratings*
+ (1—piece) + (1—ID) + ar1(bars + 0—piece:ID)

(3) *Pupil size* ∼ *Harmonic complexity ratings* + *Expressive difficulty ratings*
+ *Technical difficulty ratings*
+ (1—piece) + (1—ID) + ar1(bars + 0—piece:ID)

(4) *Pupil size* ∼ *Harmonic complexity ratings* + *Expressive difficulty ratings*
+ *Technical difficulty ratings* + *Quantity of Head Motion*
+ (1—piece) + (1—ID) + ar1(bars + 0—piece:ID)

Model 2 (including arm motion)

(1) *Pupil size* ∼ *Harmonic complexity ratings*
+ (1—piece) + (1—ID) + ar1(bars + 0—piece:ID)

(2) *Pupil size* ∼ *Harmonic complexity ratings* + *Expressive difficulty ratings*
+ (1—piece) + (1—ID) + ar1(bars + 0—piece:ID)

$$\textit{Pupil size} \sim \textit{Harmonic complexity ratings} + \textit{Expressive difficulty ratings} + \textit{Technical difficulty ratings} + (1\text{—piece}) + (1\text{—ID}) + \text{ar1(bars} + 0\text{—piece:ID)} \tag{3}$$

$$\textit{Pupil size} \sim \textit{Harmonic complexity ratings} + \textit{Expressive difficulty ratings} + \textit{Technical difficulty ratings} + \textit{Quantity of Arm Motion} + (1\text{—piece}) + (1\text{—ID}) + \text{ar1(bars} + 0\text{—piece:ID)} \tag{4}$$
